# Experimental Models of Metabolic Dysfunction-Associated Steatotic Liver Disease: A Comparative Analysis of a Choline-Deficient and Cholesterol-Enriched Diet in Rats

**DOI:** 10.3390/ijms27104230

**Published:** 2026-05-09

**Authors:** Vladimir A. Shipelin, Nikita A. Petrov, Nikita V. Trusov, Yuliya S. Sidorova, Yulia M. Markova, Zakhar A. Chalyy, Anton D. Konev, Anastasiya S. Balakina

**Affiliations:** Federal Research Centre of Nutrition and Biotechnology, 109240 Moscow, Russia; petrov-nikita-y@mail.ru (N.A.P.); nikkitosu@yandex.ru (N.V.T.); sidorovaulia28@mail.ru (Y.S.S.); yulia.markova.ion@gmail.com (Y.M.M.); tokka66@bk.ru (Z.A.C.); konev@ion.ru (A.D.K.); balakina.a.s@yandex.ru (A.S.B.)

**Keywords:** nonalcoholic fatty liver disease, NAFLD, MASLD, NASH, MASH, cholesterol-enriched diet, choline deficiency, hepatic steatosis, insulin sensitivity, oxidative stress, gene expression, cytokines, rat model

## Abstract

Metabolic dysfunction-associated steatotic liver disease (MASLD) is a widespread pathology requiring adequate preclinical models for studying pathogenesis and evaluating therapeutic and preventive agents. This study compared differential markers of MASLD pathogenesis in rats using two distinct dietary models: a choline-deficient high-fat diet (HFD-CD) and a cholesterol-enriched high-fat diet (HFD+CHOL). Male Wistar rats were fed either a control AIN93M diet, HFD-CD (40% fat, 20% fructose, and choline deficiency), or HFD+CHOL (40% fat, 20% fructose, and 1% cholesterol) for 56 days. Comprehensive assessment included phenotypic, biochemical, hematological, histomorphological parameters, oxidative stress markers, hepatocyte apoptosis, cytokine levels, and hepatic gene expression. HFD-CD induced steatosis with moderate insulin resistance, increased malondialdehyde levels, and suppressed *Acaca*, *Scd* and *ChREBP* gene expression. In contrast, HFD+CHOL caused macrovesicular steatosis, inflammation, early fibrosis, atherogenic dyslipidemia, intrahepatic cholesterol accumulation, hepatocyte apoptosis, upregulated *Srebf1*, *Cyp7a1*, and *Nfkb1* expression, and activated Nrf2-dependent antioxidant responses. HFD-CD and HFD+CHOL induce two pathogenetically distinct MASLD phenotypes. The HFD-CD model, characterized by steatosis and oxidative stress without pronounced inflammation or fibrosis, is preferable for studying the preventive potential of bioactive food compounds. Conversely, the HFD+CHOL model with inflammatory and fibrotic components is more suitable for evaluating therapeutic agents aimed at mitigating inflammation, restoring cholesterol homeostasis, and attenuating fibrosis.

## 1. Introduction

Metabolic dysfunction-associated steatotic liver disease (MASLD) (early—non-alcoholic fatty liver disease, NAFLD) is the most common cause of chronic liver disease, affecting approximately a quarter of the world’s adult population [1,2]. The pathophysiological manifestations of this disease range from simple steatosis to a progressive inflammatory process known as metabolic dysfunction-associated steatohepatitis—MASH (early—non-alcoholic steatohepatitis, NASH), which is characterized by a combination of fatty degeneration, inflammatory infiltration, and hepatocyte damage (ballooning) [3,4]. The pathogenetic transition from steatosis to MASH determines the further course of MASLD, since it is the progression of MASH that leads to the development of cirrhosis and, as a consequence, liver failure and hepatocellular carcinoma [5,6]. The high prevalence and severity of MASLD consequences determine the feasibility of developing strategies for the dietary prevention of this pathology, including the use of foods for special dietary usage and bioactive food compounds (BACs) containing components with efficacy proven in preclinical and clinical studies [7]. At the same time, the translation of experimental data into clinical practice is difficult due to the adequacy of the correspondence of preclinical models to the complex pathogenesis of MASLD in humans [8].

Existing in vivo models of MASLD are usually divided into diet-induced, genetic, and combined; however, apparently, none of them reproduces the entire possible spectrum of manifestations of this pathology in humans [9,10]. High-fat models induce obesity and insulin resistance but promote mild steatosis and inflammation in rats [11]. At the same time, methionine-choline-deficient models rapidly develop severe steatohepatitis and fibrosis, accompanied by weight loss and the absence of signs of metabolic syndrome, which limits their translatability [12,13]. Choline-deficient high-fat models allow the induction of steatohepatitis with signs of metabolic dysregulation in mice [14]. The reason for this is the disruption of one of the main choline physiological functions during its deficiency—participation in the synthesis of phosphatidylcholine, the main component of very low-density lipoproteins (VLDL) membrane, which ensures the export of triglycerides from the liver [15]. Impaired phosphatidylcholine synthesis and VLDL secretion, as a consequence, lead to accumulation of triglycerides, oxidative stress and lipotoxicity [16,17].

Excess cholesterol in the diet has a similar effect but a different mechanism [18]. In rodents, excess cholesterol in a high-fat diet increases its intrahepatic accumulation together with triglycerides, leading to inflammation, hepatic stellate cell activation (HSC), and fibrosis, thus significantly increasing the MASLD activity score. [19,20]. It has been established that a diet with only 1.25% added cholesterol against the background of an elevated fat and carbohydrate content causes severe steatohepatitis with pronounced fibrosis and impaired autophagy in rats [21]. A relatively recent review of rat models of MASLD/MASH noted that a high-fat diet with added cholesterol induces severe MASH and fibrosis more rapidly than a high-fat diet without cholesterol, with similar metabolic shifts, which is also confirmed in the study by Mustika S. et al. [11,22]. The studies [8,23,24] noted that cholesterol-enriched and choline-deficient diets have different effects on the severity of steatosis, inflammation, oxidative stress, and fibrosis, as well as on the expression of lipogenesis and inflammatory response genes, which makes them more compatible with the pathological features of MASLD/MASH in humans.

Thus, cholesterol-enriched and choline-deficient models induce impaired VLDL secretion through different mechanisms, allowing the study of different pathogenetic variants of MASLD. A comprehensive and systematic comparison of these models within a single study is of significant interest for the validation of diagnostic markers and understanding of universal and specific mechanisms of MASLD progression for the purposes of preclinical studies of therapeutic agents and promising BACs.

In this regard, the aim of this study was a comparative analysis of differential markers of MASLD pathogenesis in rats using experimental models induced by high-calorie diets with choline deficiency and the addition of 1% cholesterol, through a comprehensive assessment of phenotypic, morphometric, biochemical, hematological parameters, oxidative stress parameters, hepatocyte apoptosis and the expression of lipid metabolism and inflammatory response genes.

## 2. Results

### 2.1. Phenotypic and Morphometric Characteristics

#### 2.1.1. Body Weight Gain and Organ Weight

**Figure 1:**
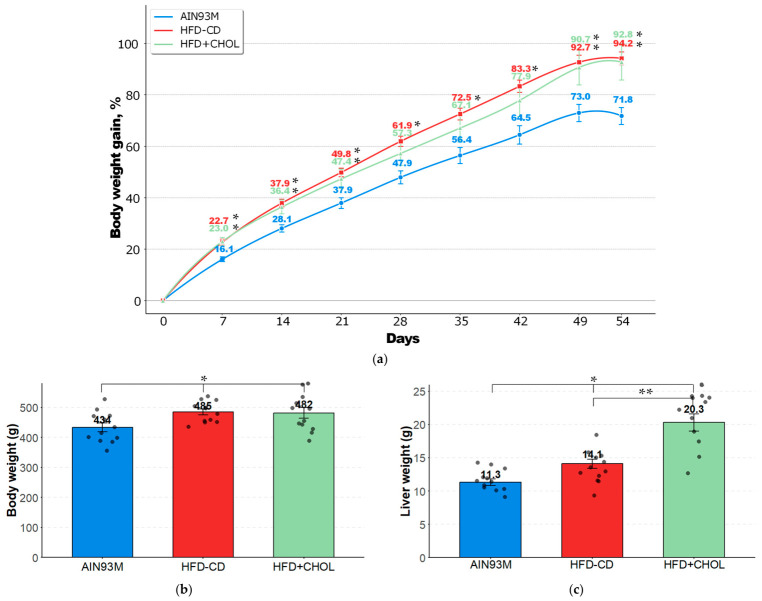
Integral indices of rats. (**a**) Body weight gain of rats from 1 to 54 days, %; (**b**) absolute body weight of rats at the end of the experiment, g; and (**c**) absolute liver weight of rats at the end of the experiment, g. *—differences are significant compared to AIN93M group (*p* < 0.05); **—differences are significant compared to HFD-CD group (*p* < 0.05). The gray circles on the figures (**b**,**c**) indicate the individual values of the animals.

Starting from the first week and throughout the experiment, consumption of a choline-deficient (HFD-CD) diet (40% fat and 20% fructose) by rats was associated with a statistically significant increase in body weight gain compared to the AIN93M control group (Figure 1a). Moreover, the maximum differences amounted to more than 30% by day 54 of the study. Animals that consumed 40% fat, 20% fructose, and an additional 1% cholesterol (HFD+CHOL) gained body weight in a similar dynamic and magnitude, with the exception of the range from 28 to 42 days, where there were no statistically significant differences with the AIN93M group. As a result, the absolute final body weight by day 54 in these groups was significantly higher compared to the control group by 12% and 11% in the HFD-CD and HFD+CHOL groups, respectively (Figure 1b).

Consumption of diets with increased quotas of fats and simple sugars and with a deficiency of choline or the addition of cholesterol also affected the liver weight of the animals. Thus, in the HFD-CD group, the absolute liver weight was significantly higher by 25%, and in the HFD+CHOL group, by 80% compared to the control group (Figure 1c). It should be noted that consumption of a diet with the addition of 1% cholesterol by rats in the HFD+CHOL group led to a significant increase in liver weight, not only compared to the control group but also compared to the HFD-CD group, exceeding the values in this group by 44%. When calculating the relative liver masses, the values were significantly higher in rats of the HFD+CHOL group, both in comparison with the AIN93M group (by 59%) and in comparison with the HFD-CD group (by 44%). In HFD-CD rats, this indicator did not differ significantly from the control values. Kidney, heart, and adrenal gland weights did not differ between groups (Table 1). An increase in the relative liver mass in rats indicates the accumulation of fat and cholesterol in hepatocytes, being the first sign of the development of steatosis in these animals, which is confirmed by quantitative assessment of fat content and histomorphological examination (see Section 2.3).

#### 2.1.2. Body Composition Based on Magnetic Resonance Relaxometry

Table 2 shows the body composition of experimental animals. As can be seen from the presented data, the body fat mass of animals in the HFD+CHOL group was significantly higher by 43% compared to the control animals AIN93M, while their lean body mass, on the contrary, was significantly lower by 5%. The total water content in the bodies of these animals was lower compared to the animals of the AIN93M group at the trend level (*p* = 0.07).

#### 2.1.3. Blood Pressure and Behavioral Indicators

The level of systolic blood pressure (BP) on day 49 in rats of all experimental groups did not differ statistically significantly and was 123.7 ± 4.5, 126.0 ± 5.0 and 134.7 ± 4.8 mmHg for the AIN93M, HFD-CD and HFD+CHOL groups, respectively.

To assess anxiety-like functions and exploratory activity in rats, the time spent in the open (OAs) and closed arms (CAs) of the elevated plus maze (EPM), the number of transitions between the arms of the maze, and the distance traveled were assessed. The evaluation results (Table 3) demonstrated the absence of a significant influence of the simulated dietary effects on the studied parameters of rats of all groups.

### 2.2. Systemic Metabolic, Hormonal and Hematological Parameters

#### 2.2.1. Insulin Resistance and Peptide Hormone Levels

**Figure 2:**
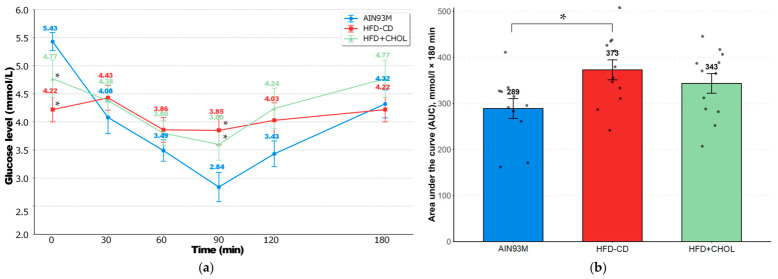
Results of ITT on the 51st day of the experiment. (**a**) Changes in glucose levels after insulin administration during 180 min; (**b**) AUC—area under curve, mmol/L × 180 min. *—differences are significant compared to AIN93M (*p* < 0.05). The gray circles on the figure (**b**) indicate the individual values of the animals.

Figure 2 shows the results of the insulin tolerance test (ITT) on day 51 of the experiment. Figure 2a shows that immediately after insulin administration, glucose levels in experimental animals decreased compared to controls by an average of 22% and 12% in the HFD-CD and HFD+CHOL groups, respectively. Moreover, despite the initially reduced levels, by the 90th minute, the glucose level in animals of these groups remained significantly higher compared to the control ones, which indicates the first signs of the development of insulin resistance. The obtained values of the area under the curve (AUC) indicate a reliable development of insulin resistance only in rats of the HFD-CD group (an increase of 29%, Figure 2b). At the same time, despite the increase in this indicator by 19% in the HFD+CHOL group, the differences were at the trend level compared to the control values (*p* = 0.09).

At the end of the experiment on day 54, glucose levels in rats in the HFD-CD group were 11% lower than control levels. The results of the enzyme-linked immunosorbent assay showed a statistically significant increase in insulin and leptin in the blood serum of rats in the HFD+CHOL group by 11% and 36% compared to the control AIN93M group, respectively. Ghrelin levels in rats of the experimental groups did not differ from those in the AIN93M control group. The data obtained are presented in Table 4.

#### 2.2.2. Blood Biochemical Parameters

The results of the determination of biochemical parameters in rat blood serum are presented in Table 5. The concentration of serum ALT in HFD-CHOL rats significantly exceeded this parameter in AIN93M animals by 31%. The concentration of alkaline phosphatase in the blood serum of rats in the HFD+CHOL group significantly exceeded the level in the control (AIN93M) by 42%. The bilirubin level in this group was 6% and 7% significantly lower than in the AIN93M and HFD-CD groups, respectively. The albumin level in rats of the HFD-CD group was 4% higher than in the control group, and in rats of the HFD+CHOL group—5% higher than in the control. The urea level in the HFD+CHOL group was 20% lower than in the AIN93M group. The concentration of uric acid in the blood plasma of rats in the HFD+CHOL group was 24% lower than in the control group and 27% lower than in rats in the HFD-CD group. The total cholesterol level in HFD-CD rats was 26% lower than the level in AIN93M, and in HFD+CHOL rats, it did not differ significantly from the control, but was significantly higher than the level in HFD-CD rats by 45%. The values of high-density lipoprotein cholesterol in rats of the HFD-CD and HFD+CHOL groups were lower by 23% and 19%, respectively, compared to the control. The level of LDL cholesterol in rats of the HFD+CHOL group was 4.2 times higher than that of the control, and 5.3 times higher than in the HFD-CD group. The triglyceride level in the HFD+CHOL group was 2 times lower than in the AIN93M group.

The urinary creatinine levels in rats of all groups did not have statistically significant differences and were 11.49 ± 1.63, 15.90 ± 2.25 and 18.17 ± 2.37 mM for the AIN93M, HFD-CD and HFD+CHOL groups, respectively. The increased creatinine values in the HFD+CHOL group relative to the AIN93M group were at the level of statistical tendency (*p* = 0.0731).

#### 2.2.3. Blood Hematological Parameters

The leukocyte formula, erythrocyte and platelet profiles of the peripheral blood of rats in the experimental groups are presented in Table 6. In the HFD+CHOL group, a significant decrease of 7% and 4% in hemoglobin content was found compared to the AIN93M HFD-CD groups, respectively. Hematocrit, red blood cell volume, and mean corpuscular hemoglobin were reduced (by 5%, 4%, and 5%, respectively) in the HFD+CHOL group compared with the AIN93M group. HFD-CD consumption in rats also caused a significant 3% reduction in mean erythrocyte hemoglobin content compared to AIN93M. No statistically significant differences were found in the leukocyte count and platelet profile in rats of the experimental groups compared to the control group.

#### 2.2.4. Serum Cytokine Levels

**Figure 3:**
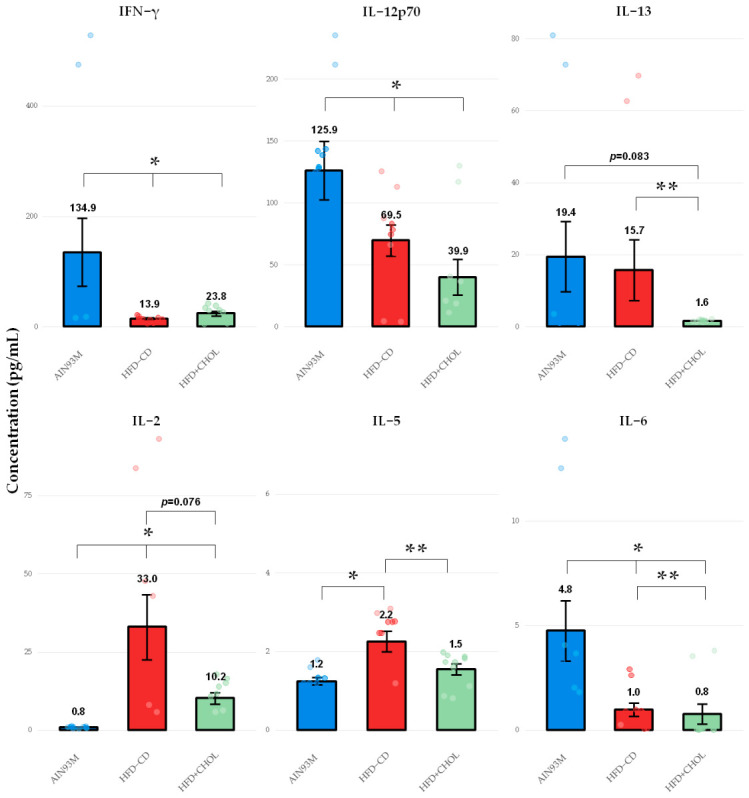
Serum levels of cytokines. *—*p* < 0.05 compared to AIN93M; **—*p* < 0.05 compared to HFD-CD. The circles on the figures indicate the individual values of the animals.

The levels of 12 cytokines were assessed in the rat blood serum: G-CSF, IFN-γ, IL-10, IL-12p70, IL-13, IL-17A, IL-1β, IL-2, IL-4, IL-5, IL-6 and TNF-α (Figure 3). Significant intergroup differences were found for IFN-γ, IL-12p70, IL-2, IL-5, and IL-6. In the HFD-CD group, compared with AIN93M, there was a 90% decrease in IFN-γ, 79% in IL-6, and 45% in IL-12p70, as well as a 41-fold increase in IL-2 and 83% in IL-5. The G-CSF level was also reduced by 69%, but these differences were only at the level of statistically significant tendency (*p* = 0.082). In the HFD+CHOL group, compared with AIN93M, the level of IFN-γ was reduced by 82%, IL-6 by 83%, and IL-12p70 by 68%, while there was a 13-fold increase in the level of IL-2. When comparing HFD+CHOL with HFD-CD, an increase in IFN-γ by 71%, a decrease in IL-2 by 69%, IL-5 by 32%, IL-6 by 20%, and IL-13 by 90% were revealed. The levels of IL-10, IL-17A, IL-1β, IL-4 and TNF-α did not show statistically significant differences between groups. Thus, a choline-deficient diet causes pronounced changes in the cytokine profile of rat blood serum: a decrease in IFN-γ, IL-6 and IL-12p70 with a significant increase in IL-2 and IL-5. Addition of cholesterol to the diet also has a significant effect on the cytokine profile of animals, although it leads to a less pronounced increase in IL-2 and a decrease in IL-13 compared to HFD-CD, with no changes in IL-5 production, which indicates different immunomodulatory mechanisms of the two models.

### 2.3. Pathological Characteristics of Liver Tissue

#### 2.3.1. Hepatic Lipid Profile

Analysis of hepatic fat, cholesterol and triglycerides content revealed statistically significant differences in the experimental groups (Table 7). Hepatic fat content of HFD-CD rats was 1.8 times higher, and of HFD+CHOL rats was 2.5 times higher compared to the AIN93M; the differences were significant. Moreover, hepatic fat content of HFD+CHOL rats was also higher in relation to the HFD-CD rats, significantly exceeding that value by 40%. Hepatic cholesterol content in HFD-CD rats was significantly higher than that of the control by almost 1.5 times, and in HFD+CHOL rats, it was 19 times higher than AIN93M and 13 times higher than in HFD-CD. The hepatic triglyceride levels in HFD-CD and HFD+CHOL rats were significantly higher than in AIN93M rats by 2.4 and 3.7 times, respectively. The obtained data indicate a significant accumulation of fat, cholesterol and triglycerides in the liver, especially in HFD+CHOL rats.

#### 2.3.2. Liver Histomorphology

**Figure 4:**
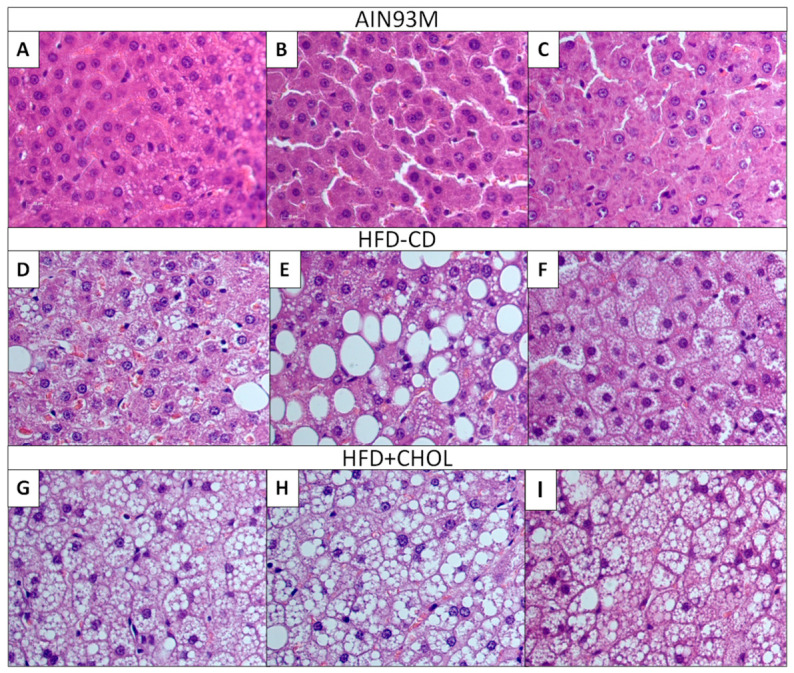
Representative microphotographs of liver sections from rats. (**A**–**C**) AIN93M; (**D**–**F**) HFD-CD; (**G**–**I**) HFD+CHOL. Hematoxylin and eosin staining. Magnification ×400.

The liver structure of the control rats is determined to be physiologically normal and consistent with the age of the animals (Figure 4A–C). The liver lobules are clearly visualized, have a regular shape, and well-defined borders. Hepatocytes are located radially around the central vein; their nuclei are centrally localized, rounded, and uniformly stained. The cytoplasm of hepatocytes contains a minimal number of vacuoles, which corresponds to normal metabolic parameters of cells. Sinusoidal capillaries are not dilated, and inflammatory infiltration is absent. Portal tracts are visualized without signs of pathological changes—bile ducts, arteries and veins are preserved. The histological picture in the control group is completely consistent with the healthy liver of animals without traces of steatosis or other pathological processes.

Feeding HFD-CD rats with a high-calorie choline-deficient diet for two months promoted the development of severe fatty liver disease (Figure 4D–F). Histologically, microvesicular and macrovesicular steatosis predominates, which is visualized as multiple vacuoles of varying sizes in the cytoplasm of hepatocytes. These vacuoles partially or completely displace the nucleus to the periphery of the cell. Some areas with light-colored cytoplasm demonstrate signs of hepatocytes ballooning—an increase in cell volume and loss of structural organization. In the portal tracts, weak inflammatory infiltration is visualized, mainly by lymphocytes and, apparently, macrophages. Signs of fibrosis and necrosis are found in isolated visual fields. Regenerative processes are limited and are represented by rare binucleated hepatocytes. Thus, the histological picture indicates the initial stage of MASLD development with a predominance of steatosis.

Histological preparations of the liver of HFD+CHOL rats, fed a diet with the addition of 1% cholesterol, are characterized by the most pronounced diffuse morphological changes in the liver (Figure 4G–I). The predominant type of steatosis is large-droplet macrovesicular, visualized as extensive vacuoles that deform the structure of hepatocytes and disrupt the architecture of the liver lobules. The sinusoids are compressed and dilated in places, which indicates a microcirculation disorder. In the portal zones, an increase in the amount of connective tissue is determined, which may suggest the development of fibrosis, although confirmation by special stains (Masson’s trichrome or Sirius red) is required for definitive assessment. In addition, significant inflammatory lymphocytic infiltrates are visualized, indicating an active process. Apoptotic bodies and binucleated hepatocytes are observed, indicating a disruption of the normal cell division cycle and the onset of a regenerative response. Thus, the histological picture corresponds to a more severe variant of MASLD, where, along with steatosis, inflammation and initial signs of fibrosis are present, which may indicate the transition of the process to the MASH stage.

#### 2.3.3. Hepatocyte Apoptosis

**Figure 5:**
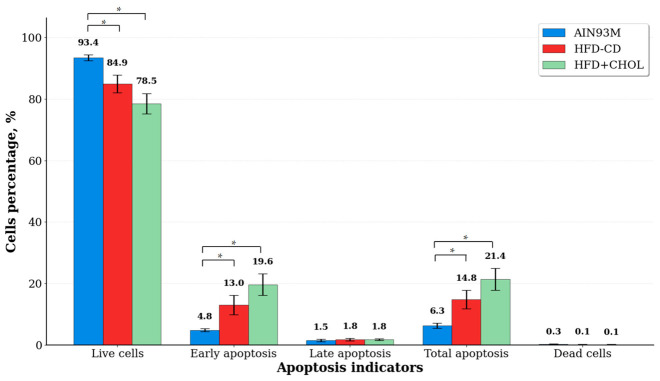
Apoptosis rates of rat hepatocytes at the end of the experiment. *—differences are significant compared to AIN93M, *p* < 0.05.

The study found that, compared to AIN93M, consumption of HFD-CD and HFD+CHOL by rats resulted in a significant decrease in the relative content of live hepatocytes (by 10% and 16%, respectively) and an increase in the proportion of cells in apoptosis (by 135% and 240%, respectively) due to the activation of the process of “early” apoptosis (Figure 5, Supplementary Table S1). No statistically significant difference was observed between the experimental parameters in HFD-CD and HFD+CHOL rats. Moreover, the number of hepatocytes in late apoptosis and the number of dead cells did not differ significantly between all groups.

### 2.4. Antioxidant Defense System

#### 2.4.1. Markers of Oxidative Damage

**Figure 6:**
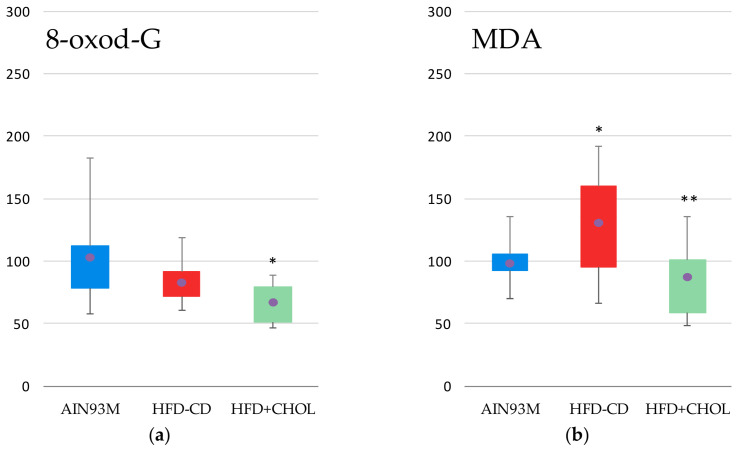
Markers of oxidative damage: (**a**) 8-oxo-2-deoxyguanosine (8-oxodG) level in rat urine and (**b**) malondialdehyde level (MDA); *—differences are significant compared to AIN93M, *p* < 0.05, **—differences are significant compared to HFD-CD, *p* < 0.05. Abscissa axis—animal groups, ordinate axis—relative concentration, % of control group. The circles on the figures indicate the mean values.

The level of the oxidative DNA damage marker 8-oxo-2-deoxyguanosine (8-oxodG) in the urine of HFD+CHOL rats was significantly lower by 35% than in the control AIN93M group. Determination of the level of malondialdehyde (MDA) in liver homogenates revealed a significant increase in this indicator in rats of the HFD-CD group compared to the control group AIN93M by 33%, while in rats of the HFD+CHOL group, this indicator did not differ significantly from the control, but was lower than the level in the HFD-CD group by 33%. Lipid peroxidation and oxidative DNA damage indices are shown in Figure 6.

#### 2.4.2. Antioxidant Protection Components

The level of reduced glutathione (GSH) in liver homogenates of HFD+CHOL rats significantly decreased by 13% compared to the control. Catalase activity and glutathione peroxidase levels in the blood serum of rats in the HFD-CD and HFD+CHOL groups did not differ from those in the AIN93M control group. However, the values in the HFD+CHOL group significantly exceeded the levels of these indicators in the HFD-CD group by 8% (catalase) and 23% (glutathione peroxidase). The results of the research are presented in Table 8.

### 2.5. Hepatic Genes Expression

#### 2.5.1. Genes of Lipid Metabolism and Cholesterol Homeostasis

**Figure 7:**
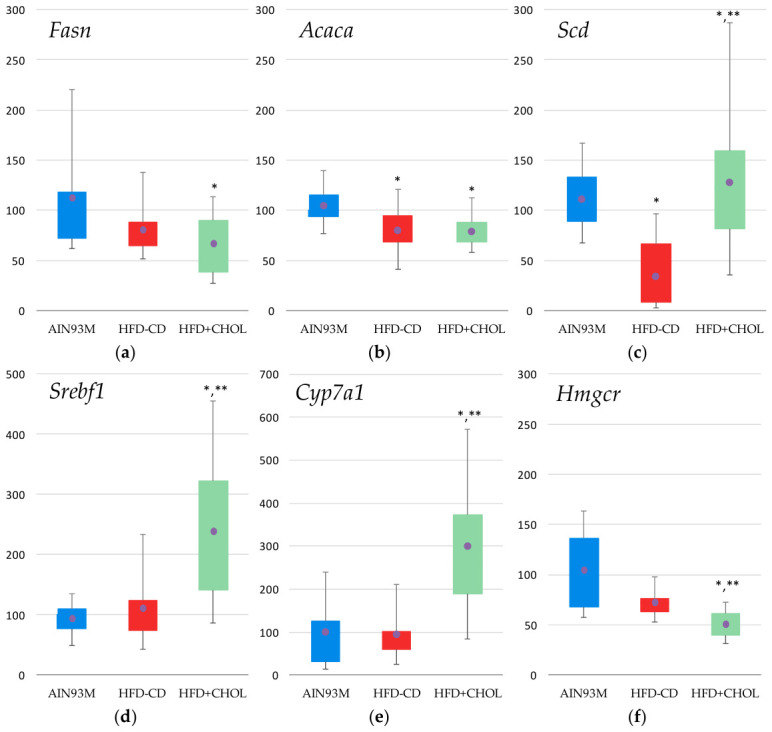
Hepatic gene expression: (**a**) *Fasn* (fatty acid synthase), (**b**) *Acaca* (acetyl-CoA carboxylase alpha), (**c**) *Scd* (stearoyl-CoA desaturase), (**d**) *Srebf1* transcription factor (sterol regulatory element-binding transcription factor 1), (**e**) *Cyp7a1* (cytochrome P450 family 7 subfamily A member 1), (**f**) *Hmgcr* (3-hydroxy-3-methylglutaryl-CoA reductase); *—differences are significant compared to AIN93M, *p* < 0.05, **—differences are significant compared to HFD-CD, *p* < 0.05. Abscissa axis—animal groups, ordinate axis—relative gene expression, % of control group. The circles on the figures indicate the mean values.

In the liver of rats from both experimental groups (HFD-CD and HFD+CHOL), real-time PCR showed a significant decrease in the expression levels of lipid metabolism enzyme genes compared to the control: *Fasn* (fatty acid synthase) by 31% in the HFD+CHOL group; *Acaca* (acetyl-CoA carboxylase alpha) by 28% and 24% in the HFD-CD and HFD+CHOL groups, respectively; *Scd* (stearoyl-CoA desaturase) in the HFD-CD group by 86% (Figure 7a–c). In the HFD+CHOL group, a more than two-fold increase in the expression of the transcription factor *Srebf1* (sterol regulatory element-binding transcription factor 1), which regulates genes associated with the formation of cholesterol and lipids, was found compared with the control group and the HFD-CD group (Figure 7d). Only in the HFD-CD group was a 20% decrease in *ChREBP* (MLX interacting protein-like) expression observed. The inclusion of excess cholesterol in the rat diet resulted in a significant 2.6-fold increase in the expression of the *Cyp7a1* enzyme gene (cytochrome P450 family 7 subfamily A member 1) in the rat liver compared to the control, which limits the synthesis of bile acids and is involved in the regulation of cholesterol levels (Figure 7e). Also, following consumption of a high-cholesterol diet, there was a 56% decrease compared to the control group in the expression of the *Hmgcr* gene, encoding 3-hydroxy-3-methylglutaryl-coenzyme A reductase, the rate-limiting enzyme in the cholesterol synthesis pathway (Figure 7f).

#### 2.5.2. Genes of Antioxidant Protection and Inflammatory Response

**Figure 8:**
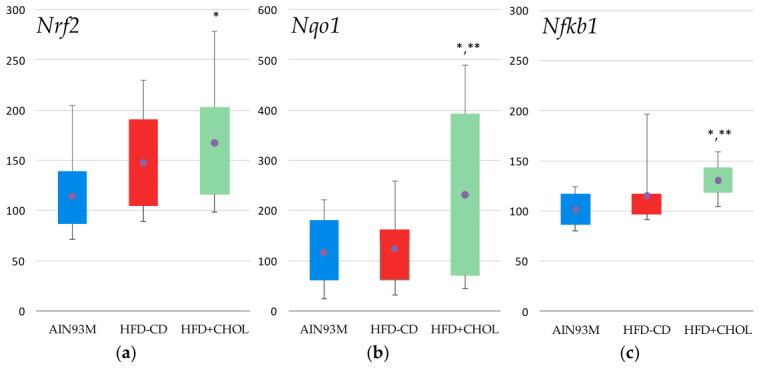
Hepatic gene expression: (**a**) *Nrf2* (NFE2-like bZIP transcription factor 2), (**b**) *Nqo1* (NAD(P)H quinone dehydrogenase 1), (**c**) *Nfkb1* (nuclear factor kappa B subunit 1); *—differences are significant compared to AIN93M, *p* < 0.05, **—differences are significant compared to HFD-CD, *p* < 0.05. Abscissa axis—animal groups, ordinate axis—relative gene expression, % of control group. The circles on the figures indicate the mean values.

When studying a number of key antioxidant defense enzymes, a reliable increase by 51% (Figure 8a) in the expression of the gene encoding the transcription factor *Nrf2* (NFE2-like bZIP transcription factor 2) was shown in the HFD+CHOL group and a 1.5- and almost two-fold increase in the expression of the gene encoding the enzyme NAD(P)H quinone dehydrogenase 1 (*Nqo1*) compared to the control (Figure 8b), although statistically insignificant. At the same time, a small significant increase was observed in the HFD+CHOL group in the expression of the gene of the anti-inflammatory nuclear transcription factor *Nfkb1* (nuclear factor kappa B subunit 1) by 29% (Figure 8c) relative to the control group and by 21% relative to the HFD-CD group, which may be a sign of the development of inflammation in the liver tissue of HFD+CHOL rats consuming a diet with the addition of 1% cholesterol. Also noteworthy is the significant 20% decrease in the expression of the catalase enzyme gene (*Cat*) in the HFD+CHOL group compared to the control, as a consequence of its increased activity noted above.

A summary table of hepatic gene expression levels of rats of all groups is presented in Supplementary Table S2.

## 3. Discussion

The obtained data demonstrate that both models used induce liver tissue disorders in rats that correspond to modern concepts of MASLD/MASH, while two pathogenetically different variants develop, each with its own advantages and disadvantages. It should be noted that the use of rats as a research subject in modeling MASLD offers its own advantages if compared to murine models, given that some mouse strains are relatively resistant to the development of fibrosis compared to rats. Moreover, Wistar rats among the studied strains exhibit a high tendency to develop steatosis [25,26,27]. The dietary modifications used in the study resulted in a statistically significant increase in body weight and absolute liver weight in both cases compared to AIN93M, but the increase in relative liver weight was significant only in the HFD+CHOL group. These results are consistent with the results of modeling high-fat and “Western” diets in rats and mice, where the combination of excess fat, simple sugars, and cholesterol causes hepatomegaly and steatosis compared to high-fat diets without cholesterol [8]. According to MRI relaxometry data, the HFD+CHOL model was accompanied by an increase in fat mass and a decrease in lean body mass, as well as an increase in circulating levels of leptin and insulin with a tendency towards higher blood pressure, indicating the formation of a systemic metabolic phenotype with obesity, hyperinsulinemia and adipokine dysregulation. A similar complex of features is characteristic of diet-induced MASH models in rats on high-fat, high-fructose, and cholesterol-rich diets. [22]. It should be taken into account that the composition of the excess fat component plays an important role in the modeling of MASLD. Thus, in the work of Buettner et al. [28], it was shown that the use of 42% fat containing lard and olive oil causes the most pronounced obesity and insulin resistance associated with liver steatosis and an increase in the activity of the sterol regulatory element-binding protein 1c transcription factor (SREBP-1c). The diet we used, with a similar excess of the fat component and a similar composition (sunflower oil instead of olive oil), also increased the activity of the transcription factor *Srebf1* (the gene encoding both isoforms of the transcription factor SREBP1) by 112%, but this effect was observed only in the HFD+CHOL group with an additional excess of cholesterol. Interestingly, in our study, according to the results of ITT, a significantly more pronounced increase in the glycemic AUC was observed in the HFD-CD group, despite normal or even reduced glucose levels and the absence of fasting hyperinsulinemia. However, the development of insulin resistance in this group is also supported by a decrease in the expression of the *ChREBP* gene, since its increased level has a positive effect on both glucose and lipid metabolism. [29,30]. It is characteristic that in the HFD+CHOL group, against the background of normal levels of *ChREBP* expression, the glycemic AUC differed from the control only at the trend level, despite elevated insulin levels and significant differences in glucose levels at the 90th minute of the test. These changes are generally consistent with the concept of high susceptibility of rats to diet-induced carbohydrate metabolism disorders, but differ from amino acid-modified models (choline or choline and methionine deficiency), which cause severe steatosis and fibrosis in the absence of obesity, hyperglycemia, and metabolic syndrome [26]. However, the observed increase in AUC while maintaining relative normoglycemia and moderate insulin levels can be considered as an early and more sensitive manifestation of decreased insulin sensitivity in the choline-deficient high-fat model, which, to our knowledge, has not previously been characterized for HFD-CD models in rats.

Marked changes in the serum lipid profile were observed in the HFD+CHOL group: a sharp increase in LDL cholesterol (>4 times compared to the control and >5 times relative to HFD-CD) with a simultaneous decrease in triglycerides and HDL. This imbalance demonstrates an atherogenic profile characteristic of MASLD progression and increased cardiovascular risk in patients [31,32]. However, rats have a lipoprotein profile that is fundamentally different from that of humans: they carry most of their cholesterol in HDL and lack active CETP (cholesterol ester transfer protein), making them resistant to atherosclerosis. LDL in rats primarily serves a transport function and becomes atherogenic, usually in the case of a combination of genetic defects and/or extreme dietary influences [33,34]. In our HFD+CHOL model, on the contrary, the level of triglycerides in the blood serum decreased, which distinguishes it from the typical atherogenic profile of patients with elevated triglycerides and reduced HDL cholesterol levels. This highlights the species-specific characteristics of lipid metabolism in rats, in which dyslipidemia when consuming a high-fat diet is not always accompanied by hypertriglyceridemia [35].

Our results demonstrate a dramatic increase in liver cholesterol content in HFD+CHOL rats (19-fold versus control and 13-fold versus HFD-CD), accompanied by a significant accumulation of total fat and triglycerides in the tissue. As noted in the introduction [19], adding cholesterol to high-fat, high-carbohydrate diets increases intrahepatic accumulation of cholesterol and triglycerides, accompanied by increased inflammation and fibrosis. Thus, excess dietary cholesterol in this combination can be considered a “second hit” component. Moreover, taking into account normal (HFD-CD) and even reduced levels (HFD+CHOL) of circulating triglycerides, the obtained data indicate predominant intrahepatic deposition of lipids and, most likely, limitation of the secretion of triglycerides with VLDL. This picture is reflected in cases described for carriers of *PNPLA3* and *TM6SF2* gene variants and on cholesterol-enriched diets in vivo, when worsening steatohepatitis is combined with relatively minor hypertriglyceridemia [18,36]. Numerous experimental and clinical studies show that free intrahepatic cholesterol, and not triglycerides per se, is the main lipotoxic molecule causing the activation of Kupffer cells and HSCs, triggering the transition from steatosis to inflammation and fibrosis with the formation of so-called cholesterol-associated steatohepatitis [36].

The histological picture in the HFD-CD group was characterized by predominantly microvesicular steatosis, with some presence of its macrovesicular manifestations, moderate portal inflammation, and isolated signs of fibrosis, which corresponds to the early stage of MASLD development. In contrast, the HFD+CHOL diet induced macrovesicular diffuse steatosis, marked inflammatory infiltration, and connective tissue accumulation in portal areas, indicating the development of a MASH-like pattern with initial fibrosis. However, given that the assessment of fibrosis was carried out only on sections stained with hematoxylin and eosin, the final conclusion about the presence and extent of fibrotic changes requires confirmation using special stains for connective tissue (Masson’s trichrome or Sirius red). Despite this, the histomorphological assessment data are quite consistent with the results of MASLD modeling in rodents, where the addition of 1–3% cholesterol to high-fat and “Western” diets (sometimes in combination with bile acids) accelerates the development of MASH and fibrosis compared to a high-fat diet without cholesterol [11,21,37,38]. Interestingly, the HFD-CD model was characterized by predominantly microvesicular steatosis (with foci of macrovesicular), while HFD+CHOL had advanced macrovesicular steatosis. In the literature, microvesicular steatosis is sometimes associated with a more severe course and oxidative stress, while macrovesicular steatosis is associated with a more “chronic” phenotype. It is possible that in a longer experiment (more than 56 days), the choline-deficient model could catch up or even surpass the cholesterol model in terms of fibrosis and inflammation. This is an important consideration for researchers planning the timing of experiments: HFD+CHOL produces a more rapid MASH-like phenotype, whereas HFD-CD may require longer timeframes to develop the full MASH pattern.

When analyzing the data on hepatocyte differentiation using flow cytometry, a decrease in the proportion of intact hepatocytes and an increase in cells in the state of “early” apoptosis were observed in both models (with no significant differences between HFD-CD and HFD+CHOL), indicating the commonality of the mechanisms of cell death in two different types of dietary damage. This is consistent with current understanding that lipotoxicity and associated forms of programmed cell death (primarily apoptosis) are essential features of progressive liver damage in MASLD/MASH. Hepatocyte apoptosis is considered one of the triggers for HSC activation and fibrogenesis: absorption of apoptotic bodies by macrophages is accompanied by the release of DAMPs and TGFβ1, which stimulate HSC and contribute to the formation of fibrosis [39,40,41]. Wiering et al. [20] note that as a result of hepatocyte death and subsequent efferocytosis, the hepatocyte–macrophage–HSC cascade determines the subsequent development of fibrosis in MASLD. The pronounced apoptosis we demonstrated in both models may reflect the initiation of such a fibrotic cascade; however, to understand the effector stage of this process, it is necessary, among other things, to take into account levels of circulating TGFβ1.

The increase in hepatic MDA levels in the HFD-CD group, taking into account normal values of this indicator in the HFD+CHOL group, against the background of severe steatosis, indicates the predominance of lipid peroxidation processes under conditions of a choline-deficient diet, which was also demonstrated in the study [17]. Moreover, Santos et al. [42] showed that when modeling choline deficiency on a normal AIN93 diet, the accumulation of triglycerides in the liver and impaired VLDL export are similarly accompanied by an increase in ROS formation, leading to an increase in LPO indices and a decrease in the activity of antioxidant defense enzymes (including catalase and superoxide dismutase). At the same time, for excess cholesterol, it has been shown that the main source of oxidative stress is free cholesterol, which accumulates in mitochondrial membranes, where it disrupts the transport of mitochondrial glutathione, increases the production of ROS, and contributes to the development of mitochondrial dysfunction. In our case, an increase in the expression of *Nrf2* and *Nqo1* genes with a simultaneous decrease in the levels of reduced glutathione and *Cat* mRNA in the liver of HFD+CHOL rats demonstrates a compensatory Nrf2-dependent antioxidant response to cholesterol-induced stress, characteristically reflecting multidirectional changes in the functioning of antioxidant genes and enzymes [43]. The decrease in urinary 8-oxodG levels in HFD+CHOL rats compared to controls in the presence of severe liver tissue stress may reflect a complex balance between ongoing oxidative DNA damage and its repair, since urinary 8-oxodG concentrations are determined not only by the rate of damage formation but also by the activity of the systems for its removal and excretion [44,45].

The HFD-CD model reflects liver lipid metabolism disorders caused by limited capacity to export triglycerides in VLDL. Moreover, a significant accumulation of triglycerides and total fat in the liver with relatively moderate changes in the systemic lipid profile is combined with a decrease in the expression of the *Acaca* and *Scd* genes, which can be characterized as a reverse regulation of de novo lipogenesis at the transcriptional level. In this group, based on normal *Srebf1* expression levels, the downregulation of lipogenic genes during liver tissue lipid overload is mediated by dysregulation of the transcription factor *ChREBP* [29,46]. At the same time, in the HFD+CHOL group, suppression of de novo lipogenesis in fat-overloaded hepatocytes occurs through a decrease in the expression of the *Fasn* and *Acaca* genes, mediated by the activation of the transcription factor *Srebf1*. When cholesterol is added to a high-fat diet, mechanisms regulating cholesterol homeostasis are also activated: a significant increase in the expression of *Cyp7a1* with a simultaneous decrease in *Hmgcr* indicates an increase in the conversion of cholesterol into bile acids and compensatory suppression of endogenous cholesterol synthesis when it is supplied in excess with the diet [47,48]. Similar transcriptional changes, expressed as a decrease in the expression of HMG-CoA reductase and LDL receptor with an increase in the expression of genes involved in the synthesis of bile acids and cholesterol export, are consistent with high-cholesterol and “Western” mouse and rat models [49,50]. The increase in *Nfkb1* in the HFD+CHOL group only indicates activation of the NF-κB pathway, is a reflection of the inflammatory process in liver tissue, and is consistent with data on the synergistic effect of excess fat and cholesterol in the induction of steatohepatitis and fibrosis [29,49,51].

Analyzing the multiplex immunoassay data, we see that both high-calorie diets significantly modified the systemic cytokine profile of rats. At the same time, we observe a rather paradoxical picture, which at first glance contradicts the severity of liver damage in the HFD+CHOL model. In the HFD-CD group, there was a significant decrease in IFN-γ and IL-12p70 (Th1-associated cytokines), as well as IL-6, with a significant increase in IL-2 and IL-5. Adding 1% cholesterol to the diet (HFD+CHOL group) led to an even more significant decrease in IFN-γ, IL-6, and IL-12p70, as well as a sharp (90%) drop in IL-13 (a Th2-associated cytokine) compared to HFD-CD. At the same time, the level of IL-5 in this group did not differ from the control against the background of a persistent increase in IL-2, although less pronounced than in HFD-CD. Thus, in the HFD+CHOL group, significant suppression of IFN-γ and IL-12p70 (Th1 response), as well as IL-13, against the background of high cholesterol accumulation in the liver, does not indicate the absence of inflammation, but rather immunosuppression caused by lipotoxicity.

Summarizing the obtained results, as clearly presented in Table 9, the HFD-CD and HFD+CHOL models form two pathogenetically different phenotypes of MASLD, differing in the direction and severity of metabolic, inflammatory, and morphological changes. The HFD-CD model reflects hepatic lipid metabolism disturbances caused by impaired export of triglycerides as part of VLDL, which leads to significant intrahepatic fat accumulation with moderate systemic metabolic changes. In contrast, the HFD+CHOL model induces a rather severe MASH-like phenotype characterized by macrovesicular steatosis, pronounced lipotoxicity and inflammation, initial signs of fibrosis, atherogenic dyslipidemia, and significant intrahepatic cholesterol accumulation with activation of associated inflammatory and fibrogenic pathways.

### Limitations of the Study

This study has a number of limitations that should be considered when interpreting the results. Fibrosis assessment was performed on sections stained with hematoxylin and eosin, so quantitative confirmation using Sirius red or Masson trichrome staining can significantly complement the picture. All gene expression data are limited to mRNA levels, so our conclusions about gene regulation only concern transcriptional changes and do not address post-transcriptional or post-translational mechanisms. Only male rats were used in the study, so given the described sex differences in the prevalence and progression of MASLD/MASH, our results may not be fully generalizable to females. The experimental diets used in the study do not fully reproduce the structure of human nutrition (for example, they do not contain sucrose, contain a standardized amount of fiber, and have differences in micronutrient composition), so the translational significance of the results for clinical practice requires careful interpretation. The duration of the experiment was 56 days, which may not be enough for the full development of fibrosis in some models. All conclusions regarding the advantages of a particular model for specific preclinical tasks are based on comparative phenotypic, biochemical, and molecular profiles obtained under the conditions of a given experiment; extrapolation to other rat strains, animal species, or longer intervention periods should be carried out with caution. Despite these limitations, our comprehensive comparative approach provides a robust methodological basis for selecting an adequate MASLD model in accordance with specific preclinical research objectives.

## 4. Materials and Methods

### 4.1. Experimental Design

**Figure 9:**
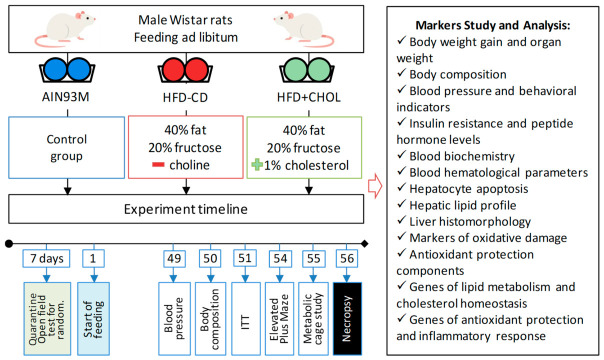
Study design.

The experiment, lasting 56 days, was carried out on male Wistar rats (with an initial body weight of ≈200 g) obtained from the nursery of the Stolbovaya branch of the Federal State Budgetary Scientific Institution “Scientific Center for Biomedical Technologies of the Federal Medical and Biological Agency”. All studies using animals were conducted in accordance with international regulations of good laboratory practice [52]. The research was approved by the Ethics Committee of the Federal State Budgetary Scientific Institution “Federal Research Center of Nutrition and Biotechnology” (protocol No. 8 dated 6 September 2024). After a 7-day quarantine, the rats were divided into 3 groups. Animals of the 1st control group (AIN93M) received the semi-synthetic diet based on AIN93M (soybean oil was replaced with a combination of sunflower and lard; dextrin and sucrose were replaced with corn starch; casein was increased to 200 g/kg, as in AIM93G; choline chloride (52% choline) was used as a source of choline instead of choline bitartrate (41.1% choline); the absence of tert-butylhydroquinone); the 2nd (HFD-CD)—a high-calorie choline-deficient diet containing 40% fat, 20% fructose and with a choline deficiency; the 3rd (HFD+CHOL)—a high-calorie diet containing 40% fat, 20% fructose and 1% cholesterol. The content of the diets used is presented in Supplementary Table S3. Animals received purified water ad libitum and food based on actual daily consumption. Rats were kept 2 per cage under artificial lighting (12 h day/night cycle), at a temperature of 22–25 °C and a relative humidity of 60–80% on a bedding of wood shavings. The general scheme of the 56-day study to model MASLD in rats is shown in Figure 9.

Systolic blood pressure in rats was measured on day 49 using a tail cuff on a non-invasive blood pressure device (ADinstruments, Dunedin, New Zealand) according to the manufacturer’s instructions.

The rats’ body composition was assessed using magnetic resonance relaxometry using the EchoMRI-1100 analyzer (EchoMRI LLC, Houston, TX, USA) on day 50 according to the manufacturer’s instructions. Parameters assessed included lean mass, fat content, and free and total water levels.

On day 51, an ITT was performed. Animals in all groups were administered insulin intraperitoneally at a dose of 0.75 U/kg. Using a OneTouch Select portable glucometer, blood glucose levels were measured before insulin administration (0 point) and after 30, 60, 120, and 180 min. Glucose curves were plotted as a function of time after insulin administration, and the area under the curve (AUC mmol/L × 180 min) was determined.

To assess the level of anxiety and exploratory activity of animals, the elevated plus maze test was carried out using the equipment, conditions, and methods described previously [53]. The rats’ movements through the maze were recorded using a Smart 3.0.04 video monitoring system (Panlab Harvard Apparatus, Barcelona, Spain). The animals were tested on day 54 during periods of minimal daily activity (from 10:00 AM to 3:00 PM). The time spent in the open and closed arms of the maze, the number of transitions between arms, and the distance traveled were recorded.

On day 55, animals were placed in metabolic cages for 16 h to collect daily urine and assess creatinine levels and oxidative DNA damage. Animals were taken out of the experiment by decapitation under light ether anesthesia on the 56th day of the experiment, after being deprived of food for 12 h. For biochemical and immunological studies, blood was collected from rats in specialized vacuum tubes; liver pieces were placed in liquid nitrogen for subsequent analysis of gene expression, MDA, and lipid levels. A piece of liver tissue was collected for histomorphological studies, after weighing the entire liver on electronic scales with an error of ±10 mg.

### 4.2. Analytical Methods

#### 4.2.1. Enzyme-Linked Immunosorbent Assay (ELISA) and Multiplex Immunoassay

Commercially available ELISA kits were used to determine the serum levels of leptin, ghrelin, insulin, catalase, and glutathione peroxidase according to the manufacturer’s instructions (Elabscience, Houston, TX, USA).

Determination of blood serum levels of cytokines (G-CSF, IFN-γ, IL-10, IL-12p70, IL-13, IL-17A, IL-1β, IL-2, IL-4, IL-5, IL-6, and TNF-α) were performed using a commercial kit (ProcartaPlex™ Rat Th-complete panel, 14plex, Invitrogen™, Thermo Fisher Scientific, Waltham, MA, USA) by multiplex immunoassay using a Luminex 200 apparatus (Luminex Corporation, Austin, TX, USA) using xMAP technology using Luminex xPONENT Version 3.1 software.

#### 4.2.2. Biochemical and Hematological Methods

The following were determined using a biochemical analyzer (Konelab 20i, Thermo Clinical Labsystems Oy, Waltham, MA, USA): in blood plasma—activity of alanine aminotransferase (ALT), aspartate aminotransferase (AST), alkaline phosphatase, content of total bilirubin, total protein, albumin, globulins, urea, uric acid, glucose, total cholesterol (TC), TC of high- (HDL) and low- (LDL) density lipoproteins, and triglycerides (TG); in urine—content of creatinine; and in fat extracted from the liver—content of TG and TC, which was calculated based on the relative mass of the liver and the amount of extracted fat. Fat was extracted from the liver using the Folch method. Samples of lyophilized liver were ground in a mortar and transferred to test tubes. A mixture of chloroform and methanol (2:1) in a ratio of 1:10 was added to the samples. The samples were stirred for 1.5 h, then distilled water was added in a volume of 1/3 the solvent volume, after which the mixture was centrifuged at 3500 rpm for 5 min. The fat dissolved in chloroform, which settled at the bottom of the test tube, was collected in pre-weighed flasks. Chloroform was then added to the test tubes at a volume of 1/3 of the original solvent volume, centrifuged at 3500 rpm for 5 min, and the chloroform-dissolved fat was transferred back into the flasks. The resulting combined fat extracts were evaporated on a rotary evaporator until all chloroform was removed. The flasks with the fat deposited on the walls were placed in a muffle furnace at 80 °C for 15 min. After cooling, the flasks were weighed, and the mass of the extracted fat was determined from the difference from the initial flask mass. Before analysis, a sample of the fat was pre-dissolved in 95% ethanol at a concentration of 2.5 mg/mL.

The hematological profile was studied on a Coulter ACT TM 5 diff OV hematology analyzer (Beckman Coulter, USA) using reagents manufactured by the same company. Hematological studies included: determination of the number of red blood cells (RBCs), white blood cells, platelets, hemoglobin (HGB), hematocrit (HCT), mean corpuscular volume (MCV), mean corpuscular hemoglobin (MCH), mean corpuscular hemoglobin concentration (MCHC), calculation of the white blood cell formula (neutrophils, eosinophils, basophils, lymphocytes, and monocytes), and determination of the mean platelet volume.

#### 4.2.3. Histological Analysis

When conducting histological studies, liver tissue samples were immediately cooled to a temperature of 0–2 °C after collection, fixed in a 3.7% formaldehyde solution in 0.1M sodium phosphate buffer, pH 7.00 ± 0.05, for at least 3 days, dehydrated in alcohols of increasing concentration, impregnated with xylene, and filled with Histomix homogenized paraffin medium on an automated block filling station. Paraffin Sections 3–4 µm thick were prepared on a Microm HM355s sled microtome (Leica, Wetzlar, Germany), stained with hematoxylin and eosin, and viewed using AxioVision Rel.4.8 software (Carl Zeiss) in an AxioImager Zl microscope (manufactured by Carl Zeiss, Oberkochen, Baden-Wuttemberg, Germany) equipped with a digital camera at a magnification of ×400.

#### 4.2.4. Flow Cytometry

The study of apoptosis of rat hepatocytes was carried out using flow cytometry. Hepatocyte suspension was obtained using an automated system for mechanical tissue homogenization, BD Medimachine (Becton Dickenson and Company, Franklin Lakes, NJ, USA). The suspension was washed once with phosphate-buffered saline (pH 7.2–7.4), and a sample with a cell concentration of 1 × 10^6^/cm^3^ was prepared. Hepatocytes were stained with fluorochrome-conjugated annexin V (AnV-FITC) and the vital dye 7-aminoactinomycin (7-AAD) (Beckman Coulter, Brea, CA, USA), followed by detection on an FC-500 flow cytometer (Beckman Coulter, Brea, CA, USA). The excised liver pieces and cell suspension were kept on ice during the work. The results are presented as the percentage of living cells and hepatocytes in different stages of apoptosis per 100,000 counted objects in each sample.

#### 4.2.5. High-Performance Liquid Chromatography (HPLC), HPLC-MS/MS, and Spectrophotometry Assays

Levels of malondialdehyde and reduced glutathione were determined in rat liver homogenates. Liver homogenization was performed at 4 °C. A 0.31 g liver sample was washed in 1.15% KCl solution and dried using filter paper. Liver homogenate was prepared in an isolation medium: 0.154 M KCl in 0.05 M Tris-HCl buffer (pH 7.4), in a ratio of 1:4 (weight:volume) in a Potter-Elvehjem homogenizer for 90 s at 1200 rpm. The volume of buffer added to the sample was 1.2 mL. The liver homogenate was stored at −80 °C until analysis.

For MDA analysis, 1.1.3.3 tetramethoxypropane (≥99%, Acros Organics, Geel, Belgium) was used as the MDA standard, as well as TBA (≥98%, Sigma, Burlington, MA, USA), H_3_PO_4_ (reagent grade, Merck, Darmstadt, Germany), sodium dodecyl sulfate (≥97%, Merck), BHT (≥99%, CDH), CH_3_OH (≥99.8%, J.T.Baker, Avantor, Radnor, PA, USA), KH_2_PO_4_ (reagent grade, Reakhim, Voskresensk, Russia), and deionized water. The reaction mixture, with a volume of 1.0 mL, including 712.5 μL of 0.1% H_3_PO_4_; 200 μL of 0.6% TBA; 60 μL of 8.1% sodium dodecyl sulfate; 27.5 μL of CH_3_OH with 125 mM BHT (the concentration of the antioxidant in the reaction mixture was 3.125 mM), was added to 100 μL of a standard MDA solution or 10-fold diluted liver homogenates. The reaction mixture was incubated for 30 min at 90 °C; cooled on an ice pad for 10 min; centrifuged at 10,000× *g* and 4 °C for 15 min; the supernatant was collected and analyzed using a HPLC system (Chromatron 1411, Labteh, Moscow, Russia). The chromatograph was equipped with an Eclipse XDB-C18 column (4.6 × 150 mm, 5 μm; Agilent, Santa Clara, CA, USA) and a fluorimetric detector RF-20A/20Axs (Shimadzu, Kyoto, Japan). Measurements were carried out at wavelengths λex = 527 nm, λem = 551 nm. The mobile phase consisted of 0.05 mM KH_2_PO_4_ (pH 6.8) and CH3OH in a ratio of 60/40, respectively; flow rate—1 mL/min; injection volume—50 μL; and analysis duration—6 min.

To determine reduced glutathione, 0.15 mL of liver homogenate (1:4), 1.35 mL of 6% TCA were added to 2 mL tubes and the mixture was centrifuged for 20 min at 4000 rpm, 4 °C. After centrifugation, 0.5 mL of the homogenate supernatant was collected in new tubes, 1 mL of 0.4 M Tris-HCl buffer with 5 mM EDTA (pH 8.8) and 0.05 mL of 0.01 M methanol solution of Ellman’s reagent were added. The mixture was vortexed, and the change in optical density (E) was recorded at a wavelength of 412 nm. Calculation of GSH μmol/g tissue: =(E × 0.00205 L)/(13,600 M^−1^ cm^−1^ × 0.01 g) × 1,000,000 = E × 15.0735.

The following sample preparation was used to determine 8-oxo-2-deoxyguanosine (8-oxodG) levels in urine: 200 µL of standard or urine solution was diluted in 200 µL of methanol, stored in a freezer at −20 °C for 1 h, centrifuged at 10,000× *g* for 10 min, and the supernatant was collected and analyzed using gradient HPLC-MS/MS analysis. Mobile phase: component A—deionized water, 0.1% HCOOH; component B—acetonitrile. Gradient: 0 min—5% B; 2 min—5% B; 12 min—20% B; 15 min—20% B; 16 min.—5% B. Analysis time is 23 min; flow rate: 0.9 mL/min; injection volume: 10 μL; column—Atlantis, C18, 4.6 × 250 mm, with a sorbent pore diameter of 5 μm. Detector type: Agilent 6410 Triple Quadrupole LC/MS; ion source: electrospray; nebulizer gas flow rate (nitrogen): 12 L/min; detection type: MRM; capillary voltage: 4000 V; and fragmentor voltage: 60 V for 8-oxo-2-deoxyguanosine. The isolated and degraded molecular weight of the parent ion for 8-oxo-2-deoxyguanosine is 284.1, and the determined daughter ion is 168.1.

#### 4.2.6. RNA Extraction and RT-qPCR

The expression of the following genes was assessed in rat liver by real-time reverse transcription polymerase chain reaction (RT-PCR): fatty acid synthase (*Fasn*), acetyl-CoA carboxylase alpha (*Acaca*), stearoyl-CoA desaturase (*Scd*), carnitine palmitoyltransferase 1A (*Cpt1a*), transcription factor peroxisome proliferator-activated receptor alpha (*Ppara*), transcription factor sterol regulatory element-binding transcription factor 1 (*Srebf1*), transcription factor MLX interacting protein-like (*ChREBP*), transcription factor NFE2-like bZIP transcription factor 2 (*Nrf2*), catalase (*Cat*), superoxide dismutase 1 (*Sod1*), glutathione peroxidase 1 (*Gpx1*), heme oxygenase 1 (*Hmox1*), NAD(P)H quinone dehydrogenase 1 (*Nqo1*), transcription factor nuclear factor kappa B subunit 1 (*Nfkb1*), cytochrome P450 family 7 subfamily A member 1 (*Cyp7a1*), and 3-hydroxy-3-methylglutaryl-CoA reductase (*Hmgcr*) were identified. Total RNA was isolated from liver tissue using the ExtractRNA reagent (Eurogen, Moscow, Russia), and complementary DNA was synthesized using the MMLV RT kit (Eurogen, Moscow, Russia) according to the manufacturer’s protocol. Real-time PCR was performed using primers and probes from DNA Synthesis LLC (Moscow, Russia). The PCR reaction mixture with a total volume of 25 μL contained 2.5 μL of cDNA (diluted 1:10) obtained in the reverse transcription reaction from 2 μg of total RNA, 2.5 μL of 10× Taq Turbo buffer for HS Taq DNA polymerase (with 2.5 mM MgCl2) (Eurogen, Moscow, Russia), 1 μL of (F+R) primers (10 μM), 0.5 μL of FAM probe (10 μM), 1.0 μL of dNTPs mixture (10 mM) (Eurogen, Moscow, Russia), 0.25 μL of HS Taq DNA polymerase (5 U/μL) (Eurogen, Moscow, Russia), and 17.25 μL of nuclease-free water (Thermo Scientific, Waltham, MA, USA). Amplification was performed on a CFX96 instrument (Bio-Rad, Hercules, CA, USA). Gene expression was assessed by the threshold cycle value and normalized relative to the reference genes *Actb* and *Gapdh* using the 2^−ΔΔCt^ method.

### 4.3. Statistical Processing of Experimental Data

Statistical processing of the obtained results was performed using the one-way analysis of variance ANOVA method using the SPSS Statistics 20 software package (IBM). The sample was tested for normality using the Kolmogorov–Smirnov test at *p* < 0.05. In case of rejection of the null hypothesis, the method of multiple comparison of means—Tukey’s test (q-test)—was used. The mean (M) and standard error of the mean (SEM) were calculated; the data are presented as M ± SEM. If the sample did not correspond to the normal distribution, the nonparametric Kruskal–Wallis test was used. In case of rejection of the null hypothesis, the multiple comparison method—Dunn’s test—was used. The median (Me), lower (Q1), and upper (Q3) quartiles were determined. The data are presented as Me (Q1–Q3). When assessing statistical differences in gene expression levels, the nonparametric Mann–Whitney U-test was used. Differences between groups of animals were considered significant at a significance level of *p* < 0.05.

## 5. Conclusions

Understanding the molecular and cellular mechanisms underlying MASH progression opens up promising targets for the development of effective therapy and prevention of MASLD. In this study, a comprehensive comparative analysis of two diet-induced MASLD models in rats was conducted, which allowed us to establish that choline-deficient (HFD-CD) and cholesterol-enriched (HFD+CHOL) diets cause different systemic metabolic shifts and morphological changes in liver tissue, forming individual molecular genetic patterns, including the expression of key genes of lipogenesis, cholesterol homeostasis, antioxidant protection and inflammatory response. It was found that the HFD+CHOL model, in contrast to HFD-CD, is characterized not only by inflammation but also by the phenomenon of systemic immunosuppression. This suggests that excess cholesterol shifts the pathogenesis of MASLD into a phase of complex immune imbalance, where systemic markers may “mask” the true severity of liver damage. Thus, the HFD+CHOL model is promising for studying therapeutic agents aimed at restoring immune homeostasis in progressive steatohepatitis. The compiled list of differential markers allows a reasonable selection of models depending on the objectives of preclinical studies. The HFD-CD model is more preferable for studying the preventive potential of dietary supplements aimed at the initial stages of overload and oxidative damage of hepatocytes. In turn, the HFD+CHOL model, which reproduces the MASH-like variant of MASLD, is more likely to be adequate for assessing the therapeutic efficacy of bioactive substances and pharmacological agents aimed at relieving the inflammatory response, correcting cholesterol homeostasis disorders, and inhibiting fibrogenesis. The obtained results complement the methodological basis for preclinical evaluation of the effectiveness of BACs for the prevention and correction of MASLD.

## Figures and Tables

**Table 1:** The weight of animal organs at the end of the experiment.

Organs	Groups		
	AIN93M	HFD-CD	HFD+CHOL
Liver (relative), %	2.63 [2.36–2.91]	2.89 [2.42–3.40]	4.17 ^1,2^ [3.70–4.95]
Kidneys, g	2.08 ± 0.08	2.21 ± 0.05	2.27 ± 0.08
Heart, g	1.25 ± 0.07	1.34 ± 0.05	1.29 ± 0.04
Adrenal glands, g	0.060 ± 0.003	0.058 ± 0.001	0.063 ± 0.002

^1^—differences are significant compared to AIN93M and ^2^—differences are significant compared to HFD-CD; *p* < 0.05.

**Table 2:** Body composition of experimental animals on the 50th day of the experiment.

Parameter	Groups		
	AIN93M	HFD-CD	HFD+CHOL
Fat mass, %	10.9 [9.5–11.7]	14.5 [10.1–17.5]	15.6 ^1^ [13.5–17.1]
Lean mass, %	82.3 ± 1.1	79.7 ± 1.3	78.3 ± 1.2 ^1^
Free water, %	0.19 [0.13–0.24]	0.21 [0.18–0.27]	0.17 [0.15–0.23]
Total water, %	69.5 ± 0.9	67.5 ± 1.1	66.6 ± 1.1

^1^—differences are significant compared to AIN93M; *p* < 0.05.

**Table 3:** Anxiety-like functions and exploratory activity of animals in the EPM test on the 54th day.

Parameter	Groups		
	AIN93M	HFD-CD	HFD+CHOL
Time in open arms, s	39.8 [23.1–81.1]	36.1 [20.1–57.5]	60.3 [20.3–84.8]
Time in closed arms, s	198.3 [151.5–242.9]	225.1 [214.2–229.5]	186.5 [141.1–238.7]
Zone transitions	19.0 [13.5–27.5]	23.0 [15.8–30.0]	24.0 [17.0–26.3]
Distance, cm	1074.1 [863.8–1221.9]	1219.1 [1115.6–1562.8]	1306.3 [977.8–1438.6]

**Table 4:** Serum levels of glucose, insulin, leptin, and ghrelin at the end of the experiment.

Parameter	Groups		
	AIN93M	HFD-CD	HFD+CHOL
Glucose, mM	6.67 ± 0.21	5.94 ± 0.17 ^1^	6.48 ± 0.19
Insulin, μU/mL	9.56 ± 0.46	8.94 ± 0.54	10.65 ± 0.48 ^2^
Leptin, pg/mL	125.4 [99.3–186.3]	151.1 [107.3–201.7]	171.0 ^1^ [162.5–283.3]
Ghrelin, pg/mL	8.05 ± 0.21	8.03 ± 0.35	8.73 ± 0.39

^1^—differences are significant compared to AIN93M, ^2^—differences are significant compared to HFD-CD; *p* < 0.05.

**Table 5:** Blood serum biochemical parameters of animals at the end of the experiment.

Parameter	Groups		
	AIN93M	HFD-CD	HFD+CHOL
AST, IU/L	114.4 ± 25.5	128.0 ± 27.6	159.6 ± 25.8
ALT, IU/L	63.6 ± 4.51	84.0 ± 12.15	83.3 ± 5.08 ^1^
AST/ALT (De Ritis ratio)	1.92 ± 0.46	2.07 ± 0.50	2.02 ± 0.37
Alkaline phosphatase, IU/L	176.5 ± 13.0	220.8 ± 13.4	250.6 ± 14.6 ^1^
Bilirubin (total), μM	6.43 ± 0.11	6.51 ± 0.09	6.05 ± 0.06 ^1,2^
Total protein, g/L	69.5 ± 1.1	69.9 ± 0.8	72.9 ± 1.2
Albumin, g/L	38.8 ± 0.5	40.5 ± 0.4 ^1^	40.6 ± 0.3 ^1^
Globulins, g/L	39.8 ± 0.8	39.0 ± 0.7	41.9 ± 1.1
Creatinine, μM	55.4 ± 1.3	54.0 ± 1.3	53.8 ± 1.2
Urea, mM	5.94 ± 0.17	5.38 ± 0.21	4.75 ± 0.17 ^1^
Uric acid, μM	81.8 ± 3.8	85.1 ± 4.5	62.3 ± 3.2 ^1,2^
Glucose, mM	6.67 ± 0.21	5.94 ± 0.17 ^1^	6.48 ± 0.19
Cholesterol (total), mM	1.87 ± 0.07	1.39 ± 0.06 ^1^	2.02 ± 0.13 ^2^
HDL cholesterol, mM	1.15 [1.03–1.25]	0.89 ^1^ [0.74–0.98]	0.93 ^1^ [0.88–1.04]
LDL cholesterol, mM	0.15 [0.10–0.17]	0.12 [0.07–0.13]	0.63 ^1,2^ [0.46–0.68]
Triglycerides, mM	1.41 ± 0.17	1.11 ± 0.15	0.72 ± 0.08 ^1^

^1^—differences are significant compared to AIN93M, *p* < 0.05; ^2^—differences are significant compared to HFD-CD, *p* < 0.05; alkaline phosphatase—HFD-CD group relative to AIN93M group at the trend level *p* = 0.0766; total protein—HFD+CHOL relative to HFD-CD group at the trend level *p* = 0.0768; globulins—HFD+CHOL group relative to HFD-CD at the trend level *p* = 0.067; urea—HFD+CHOL group compared to HFD-CD group at the trend level *p* = 0.0526.

**Table 6:** Blood hematological parameters of animals at the end of the experiment.

Parameter	Groups		
	AIN93M	HFD-CD	HFD+CHOL
RBC, 10^12^/L	7.64 ± 0.08	7.72 ± 0.13	7.58 ± 0.13
HGB, g/L	141.3 ± 1.1	137.9 ± 2.0	132.1 ± 1.5 ^1,2^
HCT, %	38.4 ± 0.5	37.6 ± 0.7	36.5 ± 0.5 ^1^
MCV, μm^3^	50.4 ± 0.5	48.8 ± 0.6	48.3 ± 0.5 ^1^
MCH, pg	18.5 ± 0.2	17.9 ± 0.2 ^1^	17.5 ± 0.2 ^1^
MCHC, g/L	367.9 ± 3.9	367.3 ± 2.2	362.3 ± 2.9
WBC,10^9^/L	9.35 ± 0.73	8.98 ± 0.80	9.53 ± 0.54
Neutrophils, %	11.4 ± 0.7	10.1 ± 0.8	10.2 ± 0.5
Eosinophils, %	1.83 ± 0.69	1.94 ± 0.30	1.43 ± 0.30
Basophils,%	0.68 ± 0.18	1.15 ± 0.18	0.95 ± 0.18
Lymphocytes, %	83.7 ± 1.4	83.9 ± 1.0	84.9 ± 0.8
Monocytes, %	2.31 ± 0.49	3.18 ± 0.14	2.49 ± 0.23
Platelets, 10^9^/L	461.8 ± 33.1	477.7 ± 28.1	467.6 ± 42.9
Mean platelet volume, μm^3^	6.74 ± 0.09	6.54 ± 0.09	6.63 ± 0.07
Plateletcrit, %	0.31 ± 0.02	0.31 ± 0.02	0.31 ± 0.03

^1^—differences are significant compared to AIN93M, *p* < 0.05; ^2^—differences are significant compared to HFD-CD, *p* < 0.05.

**Table 7:** Fat percentage, cholesterol, and triglyceride content in fat extracted from the liver.

Parameter	Groups		
	AIN93M	HFD-CD	HFD+CHOL
Fat, %	23.7 [21.4–26.6]	42.9 ^1^ [28.6–55.2]	60.2 ^1,2^ [57.7–63.0]
Cholesterol, mg/g of liver	4.1 [3.7–4.9]	6.0 ^1^ [4.8–12.2]	78.0 ^1,2^ [73.5–84.9]
Triglycerides, mg/g of liver	30.4 [23.8–36.4]	73.2 ^1^ [45.9–131.0]	112.1 ^1^ [80.5–131.5]

^1^—differences are significant compared to AIN93M, *p* < 0.05; ^2^—differences are significant compared to HFD-CD, *p* < 0.05.

**Table 8:** Parameters of the antioxidant defense system: in the liver—reduced glutathione, in the blood serum—catalase and glutathione peroxidase.

Parameter	Groups		
	AIN93M	HFD-CD	HFD+CHOL
Glutathione, μmol/g of tissue	5.69 [4.95–6.03]	5.09 [4.95–5.17]	4.98 ^1^ [4.43–5.10]
Catalase, mU/mL	2985 [2898–3020]	2886 [2823–3003]	3129 ^2^ [2944–3442]
Glutathione peroxidase, pg/mL	7.52 [6.76–7.94]	6.98 [6.66–7.76]	8.64 ^2^ [7.35–10.08]

^1^—differences are significant compared to AIN93M, *p* < 0.05; ^2^—differences are significant compared to HFD-CD, *p* < 0.05.

**Table 9:** Summary table of the magnitude and direction of changes in indicators in the modeling of MASLD in rats using HFD-CD and HFD+CHOL during a 56-day experiment.

Studied Parameter	Comparison with AIN93M, Direction (↑ or ↓) and Effect Power *	
	HFD-CD	HFD+CHOL
Integral parameters		
▪ Body weight	↑	↑
▪ Body weight gain	↑	↑
▪ Liver weight	↑	↑↑
▪ Relative liver weight	–	↑↑
Body composition		
▪ Fat content	–	↑
▪ Lean mass	–	↓
Insulin sensitivity and blood peptide hormones		
▪ Insulin sensitivity (AUC)	↓	–
▪ Glucose	↓	–
▪ Insulin	–	↑
▪ Leptin	–	↑
Blood biochemical parameters		
▪ Alkaline phosphatase	–	↑
▪ Bilirubin	–	↓
▪ Albumin	↑	↑
▪ Urea	–	↓
▪ Uric acid	–	↓
▪ Cholesterol (total)	↓	–
▪ HDL cholesterol	↓	↓
▪ LDL cholesterol	–	↑↑↑
▪ Triglycerides	–	↓
Hematological parameters (red blood cells)		
▪ Hemoglobin concentration	–	↓
▪ Hematocrit	–	↓
▪ Mean corpuscular volume of RBC	–	↓
▪ Mean corpuscular hemoglobin	↓	↓
Liver lipid profile		
▪ Fat	↑↑	↑↑↑
▪ Cholesterol	↑	↑↑↑↑
▪ Triglycerides	↑↑↑	↑↑↑
Hepatocyte apoptosis		
▪ AnV-FITC-/7-AAD- intact cells	↓	↓
▪ AnV-FITC+/7-AAD- “early” apoptosis	↑↑↑	↑↑↑
▪ Number of cells in apoptosis	↑↑↑	↑↑↑
Parameters of lipid peroxidation, DNA damage and antioxidant defense system		
▪ Malondialdehyde (liver)	↑	–
▪ 8-oxodG (urine)	–	↓
▪ Liver non-protein thiols (glutathione)	–	↓
▪ Glutathione peroxidase (blood)	–	↑
Hepatic gene expression		
▪ *Fasn* (fatty acid synthase)	–	↓
▪ *Acaca* (acetyl-CoA carboxylase alpha)	↓	↓
▪ *Scd* (stearoyl-CoA desaturase)	↓↓	–
▪ *Srebf1* (sterol regulatory element-binding transcription factor 1)	–	↑↑↑
▪ *ChREBP* (MLX interacting protein-like)	↓	–
▪ *Nrf2* (NFE2-like bZIP transcription factor 2)	–	↑↑
▪ *Cat* (catalase)	–	↓
▪ *Nfkb1* (nuclear factor kappa B subunit 1)	–	↑
▪ *Cyp7a1* (cytochrome P450 family 7 subfamily A member 1)	–	↑↑↑
▪ *Hmgcr* (3-hydroxy-3-methylglutaryl-CoA reductase)	–	↓↓
Serum cytokines		
▪ IFN-γ	↓↓	↓↓
▪ IL12p70	↓	↓↓
▪ IL2	↑↑↑↑	↑↑↑↑
▪ IL5	↑↑	↑
▪ IL6	↓↓	↓↓

Note: *—arrows ↑ and ↓ show the direction of changes in parameters; “↑” or “↓”— increase/decrease in parameter until 50% of control values; “↑↑” or “↓↓”—increase/decrease in parameter from 51% to 100% of control values; “↑↑↑”—increase in parameter from 101% to 1000% of control values; “↑↑↑↑”—increase in parameter by more than 1000% of control values; and “–”—no changes detected.

## Data Availability

Data available on request due to restrictions, e.g., privacy or ethical.

## Supplementary Materials

The following supporting information can be downloaded at: https://www.mdpi.com/article/10.3390/ijms27104230/s1.

## Abbreviations

The following abbreviations are used in this manuscript:

8-oxodG	8-oxo-2-deoxyguanosine
Acaca	acetyl-CoA carboxylase alpha
ALT	alanine aminotransferase
AST	aspartate aminotransferase
AUC	area under the curve
BACs	bioactive compounds
BP	blood pressure
CAs	closed arms
Cat	catalase
CETP	cholesterol ester transfer protein
ChREBP	MLX interacting protein-like
Cpt1a	carnitine palmitoyltransferase 1A
Cyp7a1	cytochrome P450 family 7 subfamily A member 1
DAMPs	damage-associated molecular patterns
EDTA	ethylenediaminetetraacetic acid
ELISA	enzyme-linked immunosorbent assay
EPM	elevated plus maze
Fasn	fatty acid synthase
G-CSF	granulocyte colony-stimulating factor
Gpx1	glutathione peroxidase 1
GSH	reduced glutathione
HCT	hematocrit
HDL	high-density lipoprotein
HFD+CHOL	cholesterol-enriched high-fat diet
HFD-CD	choline-deficient high-fat diet
HGB	hemoglobin
Hmgcr	3-hydroxy-3-methylglutaryl-CoA reductase (same as HMG-CoA reductase)
Hmox1	heme oxygenase 1
HPLC	high-performance liquid chromatography
HPLC-MS/MS	high-performance liquid chromatography–tandem mass spectrometry
HSC	hepatic stellate cell
IFN-γ	interferone-γ
IL	interleukin
ITT	insulin tolerance test
LDL	low-density lipoprotein
LPO	lipid peroxidation
MASH	metabolic dysfunction-associated steatohepatitis
MASLD	metabolic dysfunction-associated steatotic liver disease
MCH	mean corpuscular hemoglobin
MCHC	mean corpuscular hemoglobin concentration
MCV	mean corpuscular volume
MDA	malondialdehyde
NAFLD	non-alcoholic fatty liver disease
NASH	non-alcoholic steatohepatitis
Nfkb1	nuclear factor kappa B subunit 1
Nqo1	NAD(P)H uinine dehydrogenase 1
Nrf2	NFE2-like bZIP transcription factor 2
OAs	open arms
PNPLA3	patatin-like domain 3, 1-acylglycerol-3-phosphate O-acyltransferase
Ppara	peroxisome proliferator-activated receptor alpha
RBCs	red blood cells
ROS	reactive oxygen species formation
RT-PCR	real-time reverse transcription–polymerase chain reaction
Scd	stearoyl-CoA desaturase
Sod1	superoxide dismutase 1
Srebf1	sterol regulatory element-binding transcription factor 1
SREBP-1c	sterol regulatory element-binding protein 1c transcription factor
TBA	thiobarbituric acid
TC	total cholesterol
TCA	trichloroacetic acid
TGs	triglycerides
TGF-β1	transforming growth factor-β1
TM6SF2	transmembrane 6 superfamily member 2
TNF-α	tumor necrosis factor α
VLDLs	very-low-density lipoproteins
